# Optimization Design of CMUT Sensors with Broadband and High Sensitivity Characteristics Based on the Genetic Algorithm

**DOI:** 10.3390/s24103155

**Published:** 2024-05-16

**Authors:** Sai Zhang, Wentao Lu, Ailing Wang, Huizi He, Renxin Wang, Wendong Zhang

**Affiliations:** 1 Institute of Ultrasonic Testing, Jiangsu University, Zhenjiang 212013, China; 2 School of Mathematical Sciences, Jiangsu University, Zhenjiang 212013, China; 3 State Key Laboratory of Dynamic Testing Technology, North University of China, Taiyuan 030051, Chinawdzhang@nuc.edu.cn (W.Z.)

**Keywords:** CMUT, MEMS devices, finite element simulation, genetic algorithm

## Abstract

In this study, we propose a method for optimizing the design of CMUT sensors using genetic algorithms. Existing CMUT sensors face frequency response and sensitivity limitations, necessitating optimization to enhance their sensing performance. Traditional optimization methods are often intricate and time-consuming and may fail to yield the optimal solution. Genetic algorithms, which simulate the biological evolution process, offer advantages in global optimization and efficiency, making them widely utilized in the optimization design of Microelectromechanical Systems (MEMS) devices. Based on the theoretical framework and finite element model of CMUT sensors, we propose a CMUT array element optimization design method based on genetic algorithms. The optimization and validation results demonstrate that we have successfully designed a broadband CMUT array element consisting of four microelements with a 1–2 MHz frequency range. Compared with a randomly arranged array element, the optimized array shows a 63.9% increase in bandwidth and a 7.5% increase in average sensitivity within the passband. Moreover, the sensitivity variance within the passband is reduced by 50.2%. Our proposed method effectively optimizes the design of high sensitivity CMUT sensors with the desired bandwidth, thereby offering significant reference value for the optimization design of CMUT sensors.

## 1. Introduction

CMUT (Capacitive Micromachined Ultrasonic Transducer) sensors, derived from MEMS processing technology, represent miniaturized ultrasonic sensors with broad application prospects [1]. The inception of CMUTs on silicon wafers, utilizing silicon surface micromachining technology, dates back to 1994 when Matthew I. Haller developed the first prototype, drawing inspiration from traditional piezoelectric ultrasonic sensors [2]. In contrast to their piezoelectric counterparts, CMUT sensors offer inherent advantages such as a straightforward structure and reduced manufacturing costs, which have propelled them into the spotlight of research and garnered widespread attention [3]. As micro/nano technology advances, CMUT sensors have found diverse applications, including medical imaging, chemical sensing, and airborne applications. In medical imaging, CMUT sensors find utility in techniques such as echocardiography and ultrasound contrast imaging, delivering benefits such as superior imaging resolution and robust tissue penetration capabilities [4]. In chemical sensing, CMUT sensors boast high sensitivity and non-contact detection capabilities, rendering them suitable for gas or liquid detection tasks and conducting research on chemical reaction kinetics [5,6]. In airborne applications, CMUT sensors present advantages such as lightweight construction and seamless integration, facilitating their deployment in aerial ranging, human–machine interaction, and many other applications [7,8].

However, individual CMUT microelements exhibit limitations compared with piezoelectric sensors, including insufficient bandwidth and sensitivity. Consequently, aggregating multiple CMUT microelements into arrays is often imperative to bolster their sensing performance. Within arrays comprising multiple CMUT microelements, a trade-off relationship emerges between bandwidth and sensitivity. Employing multiple identical CMUT microelements to construct an array heightens its sensitivity, albeit at the expense of a relatively narrow bandwidth. Conversely, arrays composed of various CMUT microelements with distinct resonant frequencies can broaden the bandwidth but entail sacrificing a certain level of sensitivity. Thus, in the optimization design of CMUT arrays, balancing the interplay between bandwidth and sensitivity is paramount and constitutes one of the primary reasons for the existing limitations in the application of CMUT sensors.

To enhance the performance of CMUT sensors, existing research has conducted multifaceted optimization designs, primarily focusing on the materials and structures of CMUT microelements, array layouts, and analysis and optimization of nonlinear behaviors. In terms of optimizing the design of CMUT microelements, Jie Liu et al. [9] replaced the traditional silicon-based membrane in CMUTs with a composite membrane made of PMMA and graphene, which exhibits excellent mechanical and electrical properties, resulting in a quality factor of up to 34 at resonance frequency. Manzanares A O et al. [10] employed a perforated resonant cavity instead of the conventional vacuum-sealed cavity, enhancing the output response and bandwidth of CMUTs. Regarding the optimization design of array layouts, Maadi M et al. [11] achieved a broadband frequency range of 2–17 MHz by interleaving CMUT microelements with two different membrane diameters. Adelegan O J et al. [12] designed annular and spiral air-coupled CMUT arrays separately by improving the filling factor and reducing high-order modes, demonstrating through simulations and sample fabrication that the designed CMUT arrays possess broadband characteristics. In addressing the nonlinear behaviors of CMUTs, Jallouli et al. [13] derived the motion control equations for CMUT microelements with initial defection using the von Kármán plate theory, considering both mechanical and electrostatic nonlinearities. The results indicate that the initial defection affects both the static and dynamic behaviors of CMUTs, contributing to predicting the nonlinear behavior of imperfect CMUTs and adjusting their bifurcation topology. Stair et al. [14], focusing on the inherent nonlinearities of CMUTs, proposed different pulse sequences for small-signal and large-signal CMUT nonlinear imaging. For small-signal behavior, they proposed a two-pulse amplitude and phase modulation method to compensate for nonlinearity via subharmonic excitation. For large-signal behavior, they utilized *N* + 1 consecutive phase modulated transmit events to extract the *N* harmonics of the echo unaffected by CMUT nonlinearities.

However, existing optimization design methods have certain limitations. Traditional trial-and-error methods and empirical formulas suffer from low efficiency and lengthy cycles in the optimization process, failing to meet the demand for high performance of CMUT sensors. Numerical simulation-based optimization design methods require substantial computational resources and time, and are sensitive to initial parameters, making them prone to becoming stuck in local optimal solutions [15]. Furthermore, existing optimization design methods lack sufficient trade-offs when considering multiple performance indicators, making it difficult to comprehensively improve overall performance of CMUT sensors. Therefore, seeking an efficient, comprehensive, and multi-objective optimization design method has become one of the current research hotspots. The genetic algorithm, as an optimization algorithm simulating biological evolution processes, possesses global optimization capabilities and efficiency, and is widely applied in the optimization design of MEMS devices.

This paper applies a genetic algorithm to the optimization design of the CMUT sensor array element, aiming to optimize the layout of the CMUT sensor array element based on the expected bandwidth indicator, enabling it to simultaneously exhibit broadband and high sensitivity characteristics. The proposed method facilitates the direct design optimization of a CMUT sensor array element according to desired performance metrics, circumventing the laborious trial-and-error process of traditional numerical simulation methods and substantially reducing optimization costs. Moreover, unlike previous studies that focused only on improving either the bandwidth or sensitivity of CMUTs, the CMUT sensor array element designed in this paper considers both comprehensively. The proposed optimization design method has essential reference significance for future comprehensive optimization designs of CMUTs.

## 2. The Theory and Finite Element Model of CMUT Sensors

### 2.1. The Structure and Working Principle of CMUT Microelement

**Figure 1:**
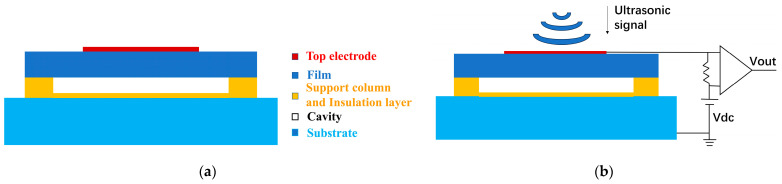
Cross-sectional diagram of CMUT microelement. (**a**) Structure of CMUT microelement; (**b**) schematic diagram of the working principle of CMUT when used as an ultrasound sensor.

The CMUT sensor, a miniaturized ultrasound sensor reliant on MEMS processing technology, necessitates a comprehensive grasp of its microelement structure and operational principles for accurate performance evaluation. Illustrated in Figure 1a is a schematic diagram delineating the microelement structure of the CMUT sensor, comprising a metal electrode, a vibrating membrane, a sealed vacuum cavity with edge support, an insulating layer, and a doped silicon substrate arranged from top to bottom [16]. In operation, the CMUT microelement functions like a parallel plate capacitor, wherein the metal electrode and silicon substrate act as the upper and lower plates, respectively, with the intervening components representing the gap between the plates. The insulating layer is pivotal in forestalling the collapse of the vibrating membrane or cavity puncture, thereby preempting a short circuit between the upper and lower electrodes. Moreover, it is a protective barrier during the sacrificial layer etching phase in the manufacturing process, shielding the device from potential damage. Incorporating a sealed vacuum within the cavity aims to diminish device damping and augment energy conversion efficiency.

The operational principle of CMUT revolves around the modulation of capacitance to facilitate the emission and reception of ultrasonic waves. When employed as a sensor, CMUT initiates by applying an initial DC bias voltage between the upper and lower electrodes to establish a balanced diaphragm state. Subsequently, as CMUT detects external ultrasonic waves, the diaphragm undergoes vibrations, altering the gap between the CMUT metal electrodes and the silicon substrate, thereby instigating a change in capacitance. This variation is then transduced into a change in current, which, following a series of signal conditioning steps, effectuates the conversion from sound signal to electrical signal, thereby enabling the reception of ultrasonic waves (refer to Figure 1b).

### 2.2. Theory Model of CMUT Microelement

The evaluation of the sensing performance of CMUT microelements encompasses various indicators, with resonant frequency (*ω*_0_) and receiving sensitivity (*S_RX_*) standing out as pivotal parameters. Theoretical derivations are indispensable to establish a solid theoretical framework for subsequent optimization endeavors.

**Figure 2:**
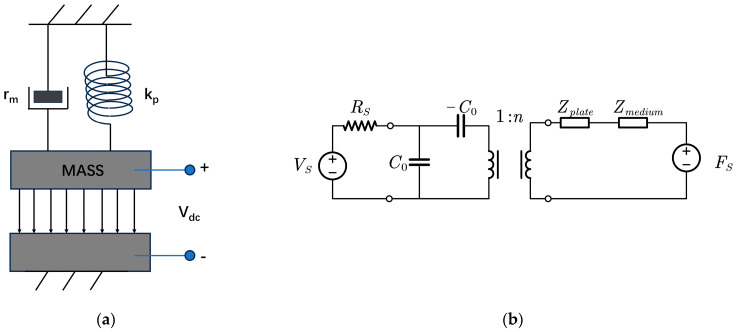
Theoretical approximation model and equivalent circuit model of CMUT microelement. (**a**) Simplified spring–mass–damper system derived from CMUT microelement; (**b**) two-port network equivalent circuit of CMUT microelement under small signal conditions.

The CMUT microelement can be conceptualized as a parallel plate capacitor featuring a movable top electrode. A mass–spring–damper system model can approximate the mechanism of this parallel plate capacitor [17], as illustrated in Figure 2a. This model incorporates spring coefficient (*k_p_*), mass coefficient (*m_p_*), and damping coefficient (*r_p_*), with damper (*r_m_*) and mass (*m_m_*) serving to simulate the impedance of the medium.

According to Ref. [18], the spring constant *k_p_* in the linear region can be obtained by the following equation:

(1) $$k_{p}=\frac{192\pi }{12a^{2}}\cdot \frac{E{t_{m}}^{3}}{1-\sigma^{2}}$$

where *E* represents Young’s modulus, *σ* represents Poisson’s ratio, and *a* represents the radius of the membrane.

The quality factor *m_p_* is obtained from the following equation [19]:

(2) $$m_{p}=1.88\pi a^{2}t_{m}$$

Under the influence of a DC bias voltage *V_dc_*, the upper electrode plate is drawn towards the lower electrode plate. Denoting the effective gap between the upper and lower electrodes as *g_eff_*, the facing area as *A*, the vacuum permittivity as *ε*_0_, and the relative permittivity of the insulating and membrane materials (assumed to be identical) as *ε_r_*, the displacement of the upper electrode plate under the combined action of electrostatic force and spring restoring force is *x_dc_*. The following equation represents the capacitance of the parallel plate capacitor:

(3) $$C_{0}=\frac{A\varepsilon_{0}\varepsilon_{r}}{g_{eff}-x_{dc}}$$

The effective air gap height *g_eff_* is computed as *(t_i_ + t_m_)/ε_r_ + g*_0_, where *g*_0_ signifies the initial gap distance without bias voltage, and *t_i_* and *t_m_* denote the insulation and membrane thicknesses, respectively. It is essential to note that, due to the presence of the oxide layer, the effective bandgap differs from the physical bandgap. Consequently, the relative permittivity is not equivalent to that in vacuum.

Based on the principle of force equilibrium:

(4) $$F_{capacitor}+F_{spring}+F_{damping}=F_{mass}$$

where the force applied by the capacitor (*F_capacitor_*) can be obtained through the principle of virtual work:

(5) $$F_{capactior}=-\frac{d}{dx}\left(\frac{1}{2}C_{0}V^{2}\right)=-\frac{1}{2}V^{2}\left[\frac{d}{dx}\left(\frac{\varepsilon_{0}\varepsilon_{r}}{g_{eff}-x}\right)\right]=\frac{\varepsilon_{0}\varepsilon_{r}AV^{2}}{2{\left(g_{eff}-x\right)}^{2}}$$

where *V* represents the total voltage across the capacitor terminals. And the spring force *F_spring_*:

(6) $$F_{spring}=-k_{p}x$$

where *k_p_* is the system’s spring constant. And the damping force *F_damping_*:

(7) $$F_{damping}=-r_{m}\frac{dx}{dt}$$

According to Newton’s second law:

(8) $$\begin{array}{c} \left(m_{p}+m_{m}\right)\frac{d^{2}x\left(t\right)}{dt^{2}}=F_{capacitor}+F_{spring}+F_{damping}\\ \;\;\;\;\;\;\;=\frac{\varepsilon_{0}\varepsilon_{r}A\left[V{\left(t\right)}^{2}\right]}{2{\left[g_{eff}-x\left(t\right)\right]}^{2}}-k_{p}x\left(t\right)-r_{m}\frac{dx}{dt} \end{array}$$

The equation in displacement *x* is nonlinear. However, for most applications, CMUT is biased by a large DC voltage (*V_dc_*) and then modulated by a small AC voltage (*V_ac_*) in transmit mode or modulated by slight pressure in receive mode (resulting in a small (*V_ac_*) induction) [20]. This assumption serves as the foundation for linearizing the static electric force of *V_dc_* and *x_dc_*. The motion equation is Taylor expanded at *x*(*t*) = 0 and linearized by neglecting higher-order terms:

(9) $$\left(m_{p}+m_{m}\right)\frac{d^{2}x\left(t\right)}{dt^{2}}+r_{m}\frac{dx\left(t\right)}{t}-\left[\frac{\varepsilon_{0}\varepsilon_{r}A{V_{dc}}^{2}}{2{\left(g_{eff}-x_{dc}\right)}^{2}}+\frac{\varepsilon_{0}\varepsilon_{r}A{V_{dc}}^{2}}{{\left(g_{eff}-x_{dc}\right)}^{3}}x\left(t\right)\right]+k_{p}x\left(t\right)=0$$

It can be simplified as:

(10) $$\left(m_{p}+m_{m}\right)\frac{d^{2}x\left(t\right)}{dt^{2}}+r_{m}\frac{dx\left(t\right)}{dt}+\left(k_{p}-\frac{\varepsilon_{0}\varepsilon_{r}A{V_{dc}}^{2}}{{\left(g_{eff}-x_{dc}\right)}^{3}}\right)x\left(t\right)=\frac{\varepsilon_{0}\varepsilon_{r}A{V_{dc}}^{2}}{2{\left(g_{eff}-x_{dc}\right)}^{2}}$$

where

(11) $$k_{s}=\frac{\varepsilon_{0}\varepsilon_{r}A{V_{dc}}^{2}}{{\left(g_{eff}-x_{dc}\right)}^{3}}$$

The phenomenon known as the spring softening effect (*k_s_*) in CMUT is contingent on the DC bias, implying that the resonant frequency of the CMUT undergoes a shift with increasing DC bias. With the application of voltage, as the top electrode approaches the bottom electrode, the electric field intensifies, prompting additional displacement of the top electrode, as though the spring constant of the top electrode decreases under the influence of the applied voltage.

Based on the formula $\omega =\sqrt{\frac{k}{m}}$, the resonant frequency can be determined:

(12) $$\begin{array}{c} \omega_{0}=\sqrt{\frac{k_{p}\;-\;k_{s}}{m_{p}\;+\;m_{m}}}\\ \;\;\;\;=\sqrt{\frac{k_{p}\;-\;\frac{\varepsilon_{0}\varepsilon_{r}A{V_{dc}}^{2}}{{\left(g_{eff}\;-\;x_{dc}\right)}^{3}}}{m_{p}\;+\;m_{m}}} \end{array}$$

From the above equation, it is evident that the resonant frequency *ω*_0_ of the CMUT is a decreasing function of the DC bias voltage *V_dc_*. This implies that the resonant frequency of the CMUT decreases as the DC bias voltage *V_dc_* increases.

When only a DC voltage is applied between the upper and lower electrodes of the CMUT, the diaphragm will strive to achieve equilibrium under the influence of the electrostatic force *F_capacitor_* and the spring force *F_spring_*. At this juncture, according to Equations (5) and (6):

(13) $$F_{capactior}=\frac{\varepsilon_{0}\varepsilon_{r}AV^{2}}{2{\left(g_{eff}-x_{dc}\right)}^{2}}=-k_{p}x_{dc}=F_{spring}$$

Therefore, it can be inferred that:

(14) $$V_{dc}=\sqrt{\frac{2k_{p}x_{dc}}{\varepsilon_{0}\varepsilon_{r}A}}\left(g_{eff}-x_{dc}\right)$$

When *V_dc_* surpasses a certain threshold, the electrostatic force exceeds the spring force, leading to the collapse of the diaphragm onto the lower electrode plate. This critical voltage is called the collapse voltage *V_coll_* and, in practical applications, the DC bias voltage should be maintained below this value. The collapse voltage can be determined by setting the derivative of Equation (14) equal to zero:

(15) $$V_{coll}=\sqrt{\frac{8k_{p}{g_{eff}}^{3}}{27\varepsilon_{0}\varepsilon_{r}A}}$$

After linearizing the parallel plate capacitor model, CMUT can be regarded as a dual-port network comprising electrical and mechanical components, depicted in Figure 2b [21]. This equivalent circuit approach proves effective in capturing the small signal behavior of CMUT, facilitating the computation of impedance and receiving sensitivity *S_RX_* as frequency functions.

In the electrical part, *C*_0_ denotes the clamping capacitance of the device under bias voltage, and −*C*_0_ signifies the spring softening capacitance. *V_s_* and *R_s_* represent the input voltage source and its resistance.

The mechanical part comprises the mechanical film and dielectric acoustic impedance. *F_s_* represents the force generated by the sound pressure source, i.e., *F_s_* = *pA*. These two components are coupled by the electromechanical transformer, envisioning the CMUT as a device that converts electrical energy into mechanical energy and vice versa. For parallel plate capacitors, the relationship between electric field, capacitance, and transformer is given by the following equation:

(16) $$E_{0}=\frac{V_{dc}}{g_{eff}-x_{dc}},C_{0}=\frac{\varepsilon_{0}\varepsilon_{r}A}{g_{eff}-x_{dc}},n=E_{0}C_{0}$$

The medium endows the sensor with impedance (*Z_a_*) for its operation, which must be incorporated into the small-signal equivalent circuit model. However, as the transducer is typically a tiny resonator compared with the wavelength (λ), *Z_a_* may substantially deviate from that of a plane wave. For a circular piston transducer with a radius of *a*, situated within an infinitely rigid plate, the acoustic impedance is given by the following equation [22]:

(17) $$Z_{a}=Z_{0}A\left[1-\frac{J_{1}\left(2ka\right)}{ka}+j\frac{H_{1}\left(2ka\right)}{ka}\right]$$

where *J*_1_ is the first-order Bessel function of the first type and *H_1_* is the first-order Struve function. *Z*_0_ represents the characteristic impedance of a plane wave (*Z*_0_ = *ρ*_0_*c*_0_), where *ρ*_0_ and *c*_0_ donate the density and velocity of sound waves in the fluid. *k* = *ω*/*c*_0_ represents the wave number. *Z_a_* possesses both a real part and an imaginary part. The real part, denoted by *r_m_*, attenuates ultrasonic waves and is referred to as radiation resistance. The imaginary part, *x_m_*, represents radiation reactance, and the resonance mass *m_m_* = *x_m_*/*ω* induces a shift in the resonance frequency.

The sensitivity of CMUT microelement can be expressed as:

(18) $$\begin{array}{c} S_{RX}=\left|\frac{I_{out}}{P_{in}}\right|=S_{RX,max}\cdot f\left(\omega \right)=\frac{nA}{r_{m}}\cdot f\left(\omega \right)\\ \;\;\;\;\;\;\;\;\;\;\;\;=\frac{V_{dc}}{{\left(g_{eff}\;-\;x_{dc}\right)}^{2}}\cdot \frac{\varepsilon_{0}\varepsilon_{r}A^{2}}{r_{m}}{\left[1+{\left(\frac{\omega }{r_{m}}\left(m_{p}+m_{m}\right)\right)}^{2}{\left(1-\frac{\omega_{0}}{\omega }\right)}^{2}\right]}^{-\frac{1}{2}} \end{array}$$

### 2.3. Finite Element Model of CMUT Microelement

**Figure 3:**
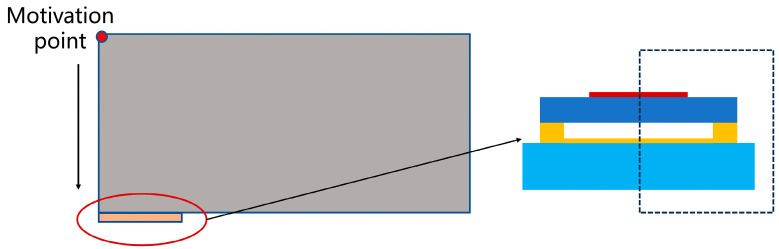
Schematic diagram of the finite element simulation model of CMUT.

To ascertain the accuracy and reliability of the theoretical derivation, finite element simulation verification of the sensing mechanism of CMUT microelement is conducted using the commercial finite element modeling software COMSOL Multiphysics (version 6.1). Given the substantial symmetry in the geometric structure, boundary loads, and constraint conditions of the CMUT microelement, a two-dimensional axisymmetric simulation model is adopted in this study, as illustrated in Figure 3, with the structural parameters delineated in Table 1.

The CMUT microelement is incorporated into the ‘Electromechanical(*eme*)’ module coupled with the ‘Solid(*solid*)’ and ‘Electrostatic(*es*)’ modules. The upper electrode and the silicon substrate are labeled as ‘Terminal 1’ and ‘Terminal 2’, respectively. Meanwhile, the lower surface and sides of the CMUT microelement are set as ‘Fixed constraints’. The CMUT microelement is placed within a domain of water material, with the environmental domain configured under the ‘Pressure acoustics, Transient(*acpr*)’ module. Additionally, the ‘Circuit(*cir*)’ module is introduced, incorporating a voltage source determined by the magnitude of the DC bias voltage (85V), with the CMUT microelement integrated into the circuit. An ‘Ammeter’ is added to measure the current passing through the CMUT microelement. For mesh generation, a free triangular mesh is employed, with the mesh in the environment domain set to ‘Extreme refinement’ and the maximum element size in the CMUT microelement region defined as one-eighth of the acoustic wavelength. Furthermore, the cavity of the CMUT microelement is specified as a ‘Dynamic mesh’ to enhance the accuracy of the simulation. In the study setup, transient analysis is executed with a time range of 0–320 ms and a time step of 1/(16 × *f_c_*), where *f_c_* denotes excitation signal frequency, utilizing the default solver. The simulation results indicate robust convergence of the model.

**Figure 4:**
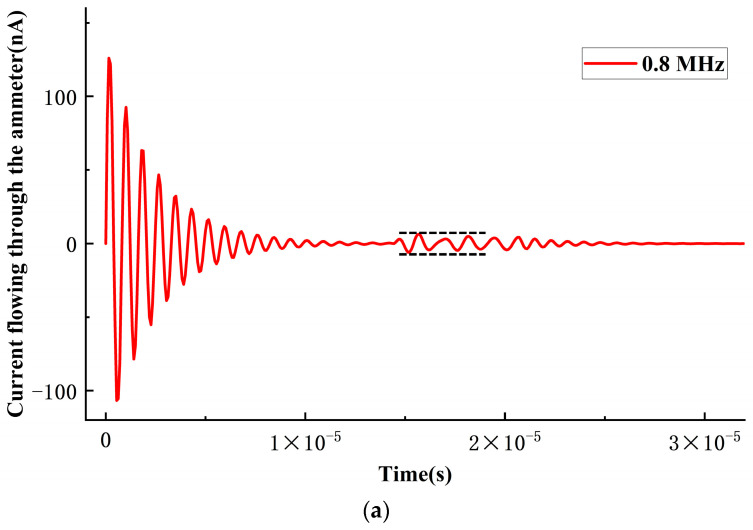

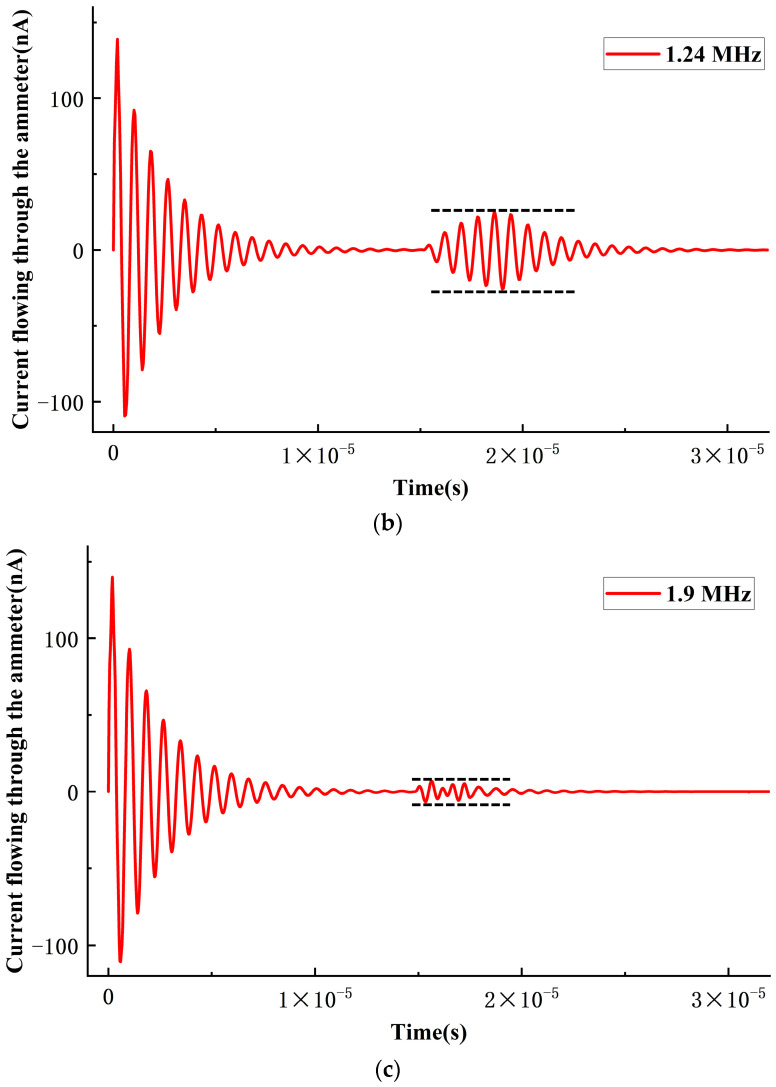
Current response under 0.8 MHz sound pressure excitation (**a**), 1.24 MHz sound pressure excitation (**b**), and 1.9 MHz sound pressure excitation (**c**). The black dashed lines indicate the amplitude variations of current response under different frequency sound pressure signals.

**Figure 5:**
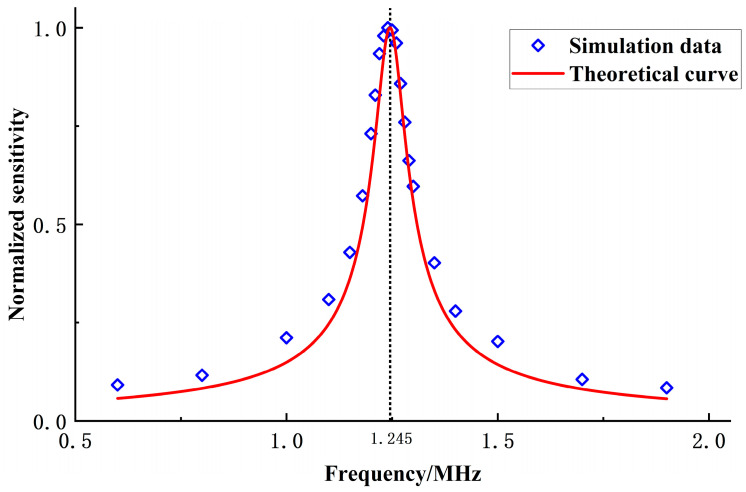
Comparison of sensitivity curves for CMUT theory and simulation.

After attaining equilibrium under the influence of a DC bias voltage, the CMUT microelement generates sinusoidal sound pressure signals with identical amplitudes but varying frequencies at the vertex within the environmental domain. This examines the current response flowing through the CMUT microelement when subjected to sound pressure signals of different frequencies. Frequency scanning is conducted using multiple sinusoidal sound pressure signals ranging from 0.6 to 1.9 MHz. Current response curves at 0.8 MHz, 1.24 MHz, and 1.9 MHz are illustrated in Figure 4a–c, respectively. The initial segment of the curve depicts the current response attributed to the DC bias voltage, while the latter portion represents the current response following the application of the sound pressure signal to the CMUT microelement. The receiving sensitivity of the CMUT microelement is determined by comparing the output current signal at various frequency ranges to the input sound pressure signal. To accomplish this, the amplitude of the current response in the latter segment under each frequency sound pressure signal is normalized by the amplitude of the input sound pressure signal and plotted as a current frequency curve. These normalized data are then juxtaposed with theoretical results, as depicted in Figure 5.

The comparison chart reveals that the resonant frequency of the CMUT microelement, as determined through finite element simulation, is 1.24 MHz. This result closely corresponds to the theoretical prediction (1.245 MHz), as evidenced by the closely matching sensitivity curve, thereby validating the accuracy of the theoretical framework. It is worth noting that potential sources of error could stem from the simplification of the CMUT microelement to a parallel plate capacitor model. Despite this simplification, the agreement between simulation and theory underscores the robustness of the theoretical framework in capturing the fundamental behavior of the CMUT microelement.

## 3. Introduction to the Genetic Algorithm and Its Application in the Optimization Design of MEMS Devices

### 3.1. Introduction to the Genetic Algorithm

**Figure 6:**
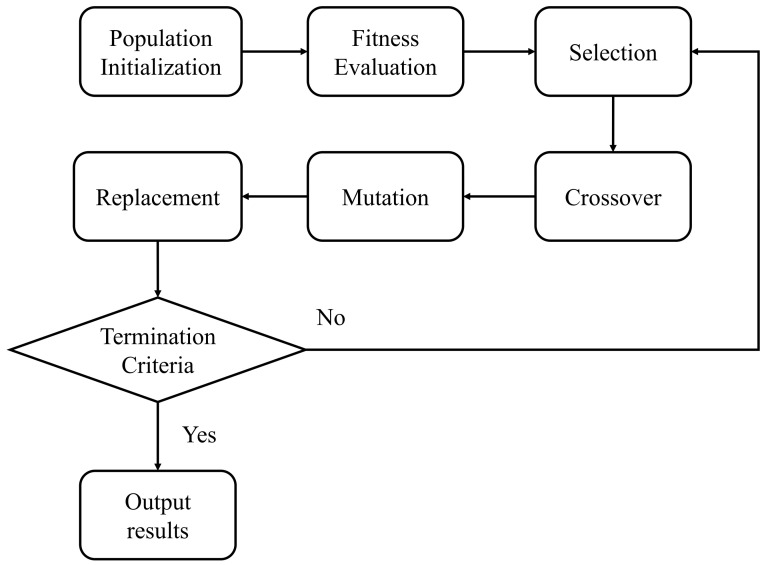
General steps of the genetic algorithm.

The Genetic Algorithm (GA) is an optimization algorithm that emulates natural selection and genetic mechanisms, with its fundamental concept rooted in Darwin’s theory of evolution [23]. By mimicking the evolutionary process observed in nature, genetic algorithms continually iterate to seek the optimal solution, possessing the capability for global optimization and efficiency. The advantages of genetic algorithms include robust adaptability to multi-modal, multi-dimensional, and multi-objective problems, along with the ability to discover global optimal solutions within vast search spaces. Genetic algorithms can effectively surmount the challenge of local optimal solutions, showcasing strong robustness and parallelism [24]. The general steps of a genetic algorithm are illustrated in Figure 6:

(1) Population initialization: A set of initial solutions is randomly generated within the search space, constituting what is known as the population. Each individual within the population represents a potential solution to the problem at hand;
(2) Fitness evaluation: The fitness of each individual within the population is computed, reflecting the quality of the solution to the problem. The fitness function, typically tailored to the specific situation, quantifies the quality of each solution;
(3) Selection: Utilizing the fitness values of individuals, specific individuals are chosen as parents to generate the next generation. Individuals with higher fitness are more likely to be selected, mirroring the principle of ‘survival of the fittest’ observed in nature;
(4) Crossover: Two individuals are selected from the chosen parent population, and new individuals, termed offspring, are generated through a crossover operation (such as single-point crossover or multi-point crossover). This process simulates genetic crossover and recombination;
(5) Mutation: Random mutation operations are applied to individuals within the offspring, altering the values of specific genes. These mutation operations aid in introducing new gene combinations and enhancing the diversity of the population;
(6) Replacement: the newly generated offspring replace the original individuals, forming a new generation of the population;
(7) Iterative evolution process: Through repeated iterations of selection, crossover, mutation, and replacement operations, individuals within the population undergo gradual optimization. Their fitness improves continuously, leading to the eventual discovery of an approximate optimal solution to the problem;
(8) Termination criteria: The termination criteria denote when to cease the evolutionary process. These criteria can include reaching a specified number of iterations, discovering solutions that meet predetermined fitness thresholds, or reaching the end of a designated duration.

Genetic algorithms can iteratively explore the search space through these steps, gradually refining solutions and converging toward the optimal solution.

### 3.2. The Application of Genetic Algorithms in the Optimization Design of MEMS Devices

The optimization design of MEMS devices often involves complex combinations of multiple parameters. Traditional optimization methods usually struggle to find the global optimal solution, while the genetic algorithm, as a global optimization algorithm, can effectively solve this problem. Therefore, genetic algorithms have been widely used in the optimization design of some MEMS devices. Through the optimization design of MEMS devices, genetic algorithms can improve device performance to meet the requirements of different application scenarios. Jabbari M et al. [25] optimized multiple structural parameters of a piezoelectric microbeam using a genetic algorithm, increasing the power generation voltage by 59% and reducing the size of the microbeam by over 50%. Nabavi et al. [26] optimized multiple physical parameters of a MEMS-based piezoelectric energy harvester using genetic algorithms, significantly increasing the collection voltage amplitude and proposing an effective solution for maximizing power generation from a single piezoelectric chip harvester. Wang et al. [27] proposed a novel semi-automated design method based on genetic algorithms for designing MEMS devices with free-form geometric shapes. Using a MEMS accelerometer with a mechanical motion amplifier as an example, they demonstrated that the sensitivity/bandwidth product of the designed MEMS accelerometer increased by 100% compared with traditional orthogonal shape-designed devices, with sensitivity increasing by 141%.

Genetic algorithms have demonstrated substantial efficacy in the optimization design of MEMS devices. By exploring a vast array of parameter combinations, genetic algorithms can identify the global optimal solution, thereby enhancing the efficiency and precision of the optimization design process. This capability enables MEMS devices to fulfill the requirements of a broader range of application scenarios, thus fostering their utilization and advancement across various fields.

The application of genetic algorithms to optimize the performance of CMUT devices based on MEMS processing technology has gained attention among researchers. Najar et al. [28] optimized the structural parameters of individual CMUT microelements, including membrane radius, membrane thickness, cavity height, and insulation layer thickness, using genetic algorithms to enhance the electromechanical coupling efficiency of CMUTs. The results demonstrated a 40% reduction in pull-in voltage and a 25% improvement in coupling efficiency for the optimized CMUTs. Zhang et al. [29] utilized genetic algorithms to sparsely optimize CMUT arrays containing 256 microelements and combined them with Kaiser apodization to reduce artifacts, and imaging results on a breast model showcased that the sparsely optimized CMUT array could achieve improved imaging performance with the same number of microelements.

Despite these advancements, the application of genetic algorithms in CMUT sensors remains relatively limited, and current methods struggle to balance the bandwidth and sensitivity of CMUT arrays. Currently, no method is available to design and optimize high sensitivity CMUT sensors based on desired bandwidth.

## 4. Optimization Design Method of CMUT Array Element Based on the Genetic Algorithm

### 4.1. Optimizing Problem Description and Steps of the Genetic Algorithm

In the optimization design of CMUT sensors, frequency response and sensitivity emerge as pivotal performance metrics. Frequency response delineates the sensor’s efficacy across different frequencies, while sensitivity influences its capacity to detect target signals. The crux of optimization design lies in striking a balance between frequency response and sensitivity to attain broadband and high sensitivity characteristics. This study leverages the optimization design of a 2 × 2 CMUT array element as a case study to validate the feasibility of the CMUT sensor array element optimization design method based on the genetic algorithm.

**Figure 7:**
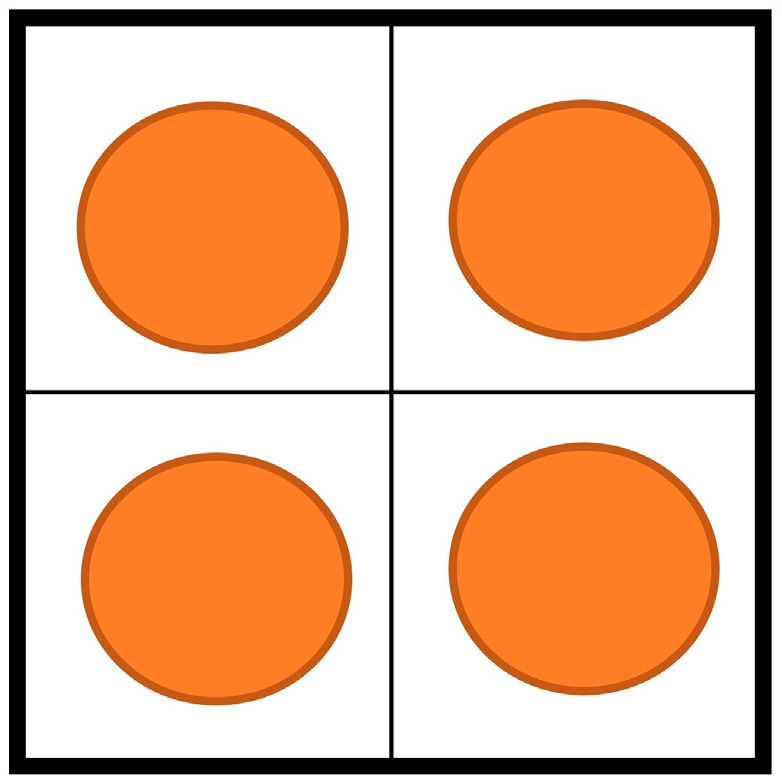
CMUT array element to be optimized.

The 2 × 2 CMUT array element slated for optimization is depicted in Figure 7. The total area of the array element is 300 μm × 300 μm, with the desired bandwidth ranging from 1 to 2 MHz. Maximizing the sensitivity of the array element within this bandwidth is paramount. With these stipulated conditions and objectives in mind, the genetic algorithm employed in this study adheres to the following specific steps:

(1) Determine the independent variables of the algorithm. Given practical process limitations, the structural parameters of various layers of materials, membrane thickness, cavity height, etc., of the CMUT microelement are typically predetermined. Under these conditions, theoretical formulas derived in Chapter 2 indicate that the resonant frequency of the CMUT microelement is solely determined by the radius of each microelement and its respective DC bias voltage. Considering practical processes, it is imperative to maintain uniformity in the DC bias voltage across microelements within the CMUT array element. Hence, the independent variables of the genetic algorithm encompass each microelement’s radius (*r*_1_, *r*_2_, *r*_3_, *r*_4_) and the uniform DC bias voltage (*V_dc_*).
(2) Determine the range of independent variables. Given that the optimized array element is divided into four regions of 2 × 2, each microelement’s radius (*r*_1_, *r*_2_, *r*_3_, *r*_4_) must be less than or equal to 75 μm. Furthermore, according to Equation (15), the uniform DC bias voltage (*V_dc_*) should be less than or equal to the minimum collapse voltage of each microelement.
(3) Determine the fitness function in the algorithm. In this case, the optimization objective is to determine the arrangement of the array element that yields the highest sensitivity based on the expected bandwidth indicator. Accordingly, the total sensitivity of the array element is selected as the fitness function to maximize its value. Within the CMUT array element, each microelement operates in parallel mode, leading to the total output current being the linear superposition of the current responses of individual microelements. Therefore, according to Equation (18), the total sensitivity of the array element is the linear superposition of the sensitivities of each microelement. Consequently, the fitness function for the genetic algorithm is as follows, where *S*_1_–*S*_4_ are computed via Equation (18):
  (19) $$S=20log_{10}\left|\frac{I_{allout}}{P_{in}}\right|=20log_{10}\left|\frac{I_{1}+I_{2}+I_{3}+I_{4}}{P_{in}}\right|=20log_{10}\left|S_{1}+S_{2}+S_{3}+S_{4}\right|$$
(4) Determine the operational parameters of the algorithm. The population size is set to 100, and the number of chromosome nodes is set to 5. For the crossover and mutation processes, the algorithm utilizes a threshold-setting approach. Both the crossover and mutation thresholds are set to 0.2. The termination criterion of the algorithm reaches a certain number of iterations, set to 100 iterations.
(5) Add additional constraints. We aim to achieve a flatter sensitivity curve for the optimized CMUT within the passband (i.e., the designated bandwidth target). Thus, minimizing the sensitivity variance within the passband as much as possible is imperative.
(6) Executing the genetic algorithm to derive optimized independent variable results and conducting corresponding finite element simulation verification.

### 4.2. Optimization Results and Finite Element Simulation Verification

**Figure 8:**
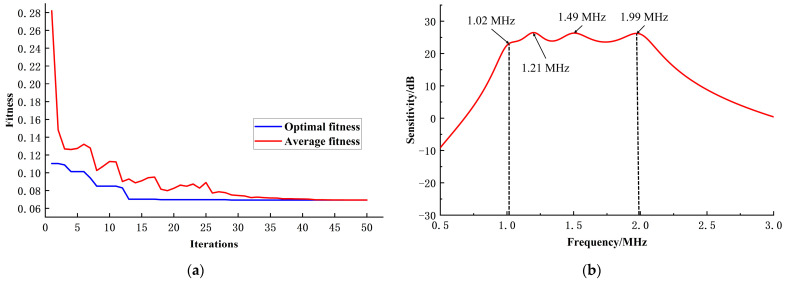
Optimization results of the genetic algorithm. (**a**) Average fitness versus optimal fitness; (**b**) sensitivity curve after optimization.

The results obtained through the optimization design of the genetic algorithm are shown in Table 2 and Figure 8.

Figure 8a illustrates that optimal fitness remains consistent across multiple generations, with the average fitness converging towards optimal fitness. This convergence indicates that the algorithm has effectively converged. Examining Figure 8b, it is evident that the passband range of the array element falls within 1–2 MHz, achieving the desired bandwidth target. Moreover, the passband exhibits a relatively flat response, meeting the optimization objective of minimizing sensitivity variance within the passband.

**Figure 9:**
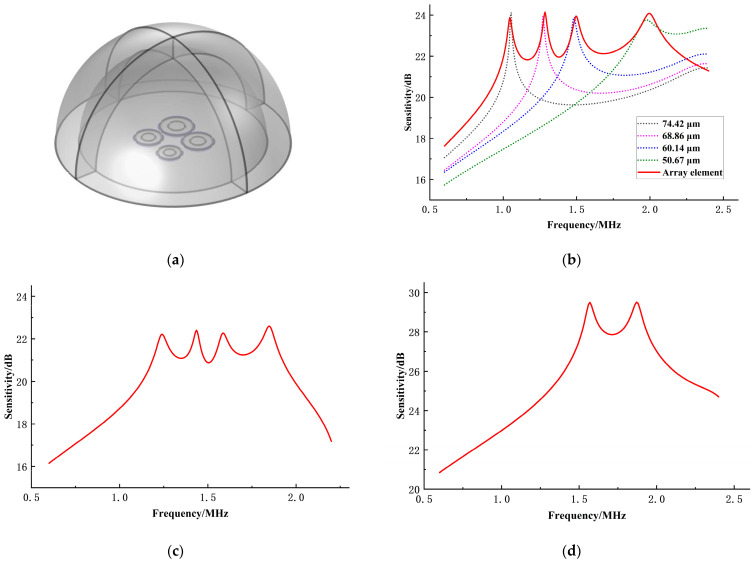
Validation of CMUT array elements using finite element simulation. (**a**) Finite element simulation model of CMUT array element in three dimensions; (**b**) finite element simulation verification of optimized results; (**c**) finite element simulation of equidistant radii microelement layout; (**d**) finite element simulation of two types of microelement layouts.

The optimization results are validated using a finite element simulation model, as depicted in Figure 9a. The finite element simulation of the CMUT array element necessitates the creation of a three-dimensional model, with each microelement’s radius set to the optimized values of *r*_1_, *r*_2_, *r*_3_, *r*_4_. The environmental domain is configured as a hemispherical region with a radius of 1000 μm, supplemented by an additional 200 μm perfect matching layer outside the environmental domain. The remaining materials and structural parameters are set consistent with Table 1. The excitation acoustic pressure signal is a sinusoidal ultrasound signal with an amplitude of 4 × 10^4^ Pa, positioned 1000 μm directly above the center of the array element. Each microelement is coupled in parallel in the ‘Circuit(cir)’ module, with the remaining physical field settings consistent with Section 2.3.

Running simulations, the sensitivity curve of the array element is shown in Figure 9b. The figure shows that the CMUT array element achieves a bandwidth of 1–2 MHz with an average sensitivity of approximately 22.8 dB within the passband, as observed in the finite element simulation. The resonance frequencies obtained from the simulations closely match the algorithm’s results and align well with those obtained from the simulations of individual microelements. The trend of the sensitivity curve obtained is mainly consistent with the optimization results of the algorithm, suggesting that the source of error may still be related to the approximation of the theoretical model.

Moreover, the optimized results are compared with other random layouts. Among them, random layouts such as equidistant radii microelement layouts (with radii of 66 μm, 61 μm, 56 μm, and 51 μm) and two types of microelement layouts (with radii of 51 μm and 56 μm) are utilized as examples, as illustrated in Figure 9c,d. The comparative analysis reveals that the array element composed of equidistant radii microelements has a passband of approximately 1.24–1.85 MHz, with an average sensitivity of about 21.2 dB within the passband. Compared with this, the optimized array element shows respective increases of 63.9% and 7.5% in bandwidth and average sensitivity. Although the array element composed of two types of microelements exhibits higher sensitivity, its bandwidth is far narrower than that achieved through the algorithmically optimized arrangement due to having only two resonance frequency peaks. Additionally, the sensitivity variance within the passband for the optimized array element is approximately 0.41, which is a 50.2% reduction compared with the array element composed of equidistant radii microelements (about 0.83). This indicates that the optimized CMUT array element demonstrates smoother and more stable characteristics within its passband, which are advantageous for ensuring the operational stability of CMUT devices in practical applications.

**Figure 10:**
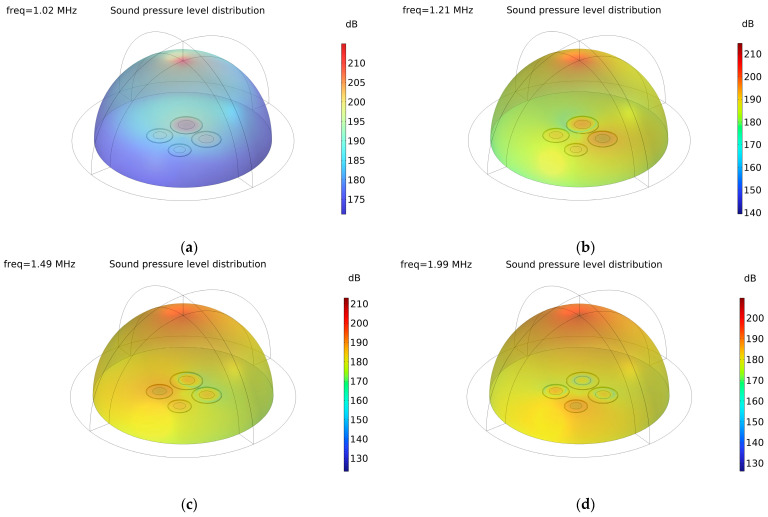
Pressure level distribution maps of the optimized CMUT array element at 1.02 MHz (**a**), 1.21 MHz (**b**), 1.49 MHz (**c**), and 1.99 MHz (**d**).

Furthermore, we have presented pressure level distribution maps of the optimized CMUT array element at various frequencies to visually elucidate the spatial and surface pressure distribution, as depicted in Figure 10a–d. Specifically, pressure level distribution maps corresponding to the resonance frequencies at the four peak positions in the sensitivity curve (1.02 MHz, 1.21 MHz, 1.49 MHz, and 1.99 MHz) are selected and plotted. For clarity, the pressure level distribution of the outer perfectly matched layer is disregarded to provide a more intuitive visualization. Observing the figures, it is apparent that, at each frequency corresponding to the peak positions, the surface pressure level of the microelement at the resonant frequency is the highest. In comparison, the surface pressure levels of other microelements at this frequency are relatively lower. This observation further underscores that the response of the CMUT array element is coupled with individual microelements.

Based on the comprehensive optimization results and finite element simulation verification data, it can be concluded that the genetic algorithm has been effectively employed to design a CMUT sensor that meets the desired bandwidth target. Consequently, the sensor’s frequency response and sensitivity have been balanced and enhanced, achieving the objectives of broadband and high sensitivity characteristics. This outcome offers more reliable technical support for utilizing CMUT sensors in various fields, such as medical imaging, chemical sensing, and airborne applications.

## 5. Conclusions

This study presents an optimization design of CMUT sensors based on the genetic algorithm to enhance their broadband and high sensitivity characteristics. By analyzing the theoretical framework and finite element model of CMUT sensors, a deep understanding of their operational principles and performance metrics is obtained. Building upon this foundation, a CMUT sensor array element optimization design method based on genetic algorithms is proposed. This method comprehensively considers the frequency response and sensitivity of the sensor, facilitating improvements in its broadband and high sensitivity characteristics. We successfully optimize the design of a broadband CMUT array element consisting of four microelements operating within the 1–2 MHz frequency range. Compared with a random array element, the optimized array element demonstrates respective increases of 63.9% and 7.5% in bandwidth and average sensitivity within the passband while reducing the variance of sensitivity within the passband by 50.2%. The results indicate that the genetic algorithm-based CMUT sensor array element design method effectively enhances the sensor’s performance, enabling the optimization design of high sensitivity CMUT sensors based on desired bandwidth criteria.

In summary, the CMUT sensor array element optimization design method employing genetic algorithms effectively enhances the sensor’s performance, yielding significant advancements in broadband and high sensitivity characteristics. This optimization approach offers crucial technical backing for the extended utilization of CMUT sensors, facilitating their broader adoption in medical imaging, chemical sensing, and airborne applications. However, it is essential to acknowledge certain limitations associated with the method proposed herein. The optimized CMUT sensor array element, devised in this study, relies on a small-signal theory model, which overlooks the inherent nonlinear characteristics of CMUTs. Further investigation is warranted to ascertain the applicability of this method to CMUTs operating in the nonlinear domain. Additionally, experimental validation utilizing corresponding CMUT samples is imperative to further corroborate the proposed method’s effectiveness and reliability.

## Figures and Tables

**Table 1:** Structural parameters of CMUT microelement in finite element simulation.

	Radius/Length (μm)	Thickness/Width (μm)	Material
Top electrode	34	0.2	Aluminium
Film	68	2.6	Doped silicon
Cavity	64	0.24	Vacuum
Insulation layer	68	0.1	Silica
Substrate	68	0.4	Doped silicon
Environment	2000	1000	Water

**Table 2:** Optimized independent variable parameters.

*r*_1_ (μm)	*r*_2_ (μm)	*r*_3_ (μm)	*r*_4_ (μm)	*V_dc_* (V)
74.42	68.86	60.14	50.67	60.55

## Data Availability

Data are contained within the article.
